# Analysis of a post-translational steroid induction system for *GIGANTEA *in Arabidopsis

**DOI:** 10.1186/1471-2229-9-141

**Published:** 2009-11-30

**Authors:** Markus Günl, Eric FungMin Liew, Karine David, Joanna Putterill

**Affiliations:** 1 Plant Molecular Sciences, School of Biological Sciences, University of Auckland, Private Bag 92019, Auckland, New Zealand

## Abstract

**Background:**

To investigate the link between the flowering time gene *GIGANTEA *(*GI*) and downstream genes, an inducible *GI *system was developed in *Arabidopsis thaliana *L. Heynh. Transgenic Arabidopsis plant lines were generated with a steroid-inducible post-translational control system for GI. The gene expression construct consisted of the coding region of the GI protein fused to that of the ligand binding domain of the rat glucocorticoid receptor (GR). This fusion gene was expressed from the constitutive cauliflower mosaic virus 35S promoter and was introduced into plants carrying the *gi-2 *mutation. Application of the steroid dexamethasone (DEX) was expected to result in activation of the GI-GR protein and its relocation from the cytoplasm to the nucleus.

**Results:**

Application of DEX to the transgenic plant lines rescued the late flowering phenotype conferred by the *gi-2 *mutation. However, despite their delayed flowering in the absence of steroid, the transgenic lines expressed predicted *GI *downstream genes such as *CONSTANS (CO) *to relatively high levels. Nevertheless, increased *CO *and *FLOWERING LOCUS T *(*FT*) transcript accumulation was observed in transgenic plants within 8 h of DEX treatment compared to controls which was consistent with promotion of flowering by DEX. Unlike *CO *and *FT*, there was no change in the abundance of transcript of two other putative *GI *downstream genes *HEME ACTIVATOR PROTEIN 3A *(*HAP3A*) or *TIMING OF CHLOROPHYLL A/B BINDING PROTEIN 1 *(*TOC1*) after DEX application.

**Conclusion:**

The post-translational activation of GI and promotion of flowering by steroid application supports a nuclear role for GI in the floral transition. Known downstream flowering time genes *CO *and *FT *were elevated by DEX treatment, but not other proposed targets *HAP3A and TOC1*, indicating that the expression of these genes may be less directly regulated by GI.

## Background

Timing the transition to flowering to synchronise with favourable seasons of the year is critical for successful sexual reproduction in many plants. *Arabidopsis thaliana *(L.) Heynh (Arabidopsis) flowers rapidly in the lengthening days of spring and summer (long days; LD 16h L/8 h dark) and shows delayed flowering in short day conditions (SD, 8 h L/16 h D) [1]. *GIGANTEA *(*GI*) is a key regulator of the photoperiodic response of Arabidopsis as plants carrying mutations in this gene no longer flower rapidly in response to LD [1,2]. Instead, the *gi *mutant develops a large rosette of leaves and thus is "gigantic" in size compared to wild type plants before finally flowering. The *gi *mutant flowers at a similarly delayed time as wild type plants in SD.

Since the role of GI in promoting flowering was first highlighted by mutant analysis [1], GI has been shown to have other distinct functions. These include roles in photomorphogenesis and in regulation of the circadian clock, an internal oscillator that regulates daily rhythms of ~24 h in duration [2-8]. A molecular basis for some of the effects of GI on clock function was recently provided [9]. GI was shown to interact with an F-box containing blue light receptor ZEITLUPE (ZTL) leading to the proteasome-dependant degradation of the central clock component TIMING OF CHLOROPHYLL A/B BINDING PROTEIN 1 (TOC1) [9,10].

A module of genes acting in the order *GI *- *CONSTANS *(*CO*) - *FLOWERING LOCUS T *(*FT*) were shown to promote flowering in LD [reviewed by [11]]. These are all rhythmically expressed and regulated by the circadian clock [11]. *FT *encodes a strong promoter of flowering which was recently shown to function as a mobile flowering hormone or "florigen" [reviewed by [12]]. After induction of *FT *transcription, FT protein was produced in the vasculature of the leaves, mobilized in the phloem and uploaded in the shoot apex where it interacted with a bZip transcription factor called FD [reviewed by [12]]. This led to activation of genes including the floral integrator *SUPPRESSION OF OVEREXPRESSION OF CO1 *(*SOC1*) in the shoot apex, then floral meristem identity genes such as *APETALA 1 *(*AP1*) and the transition from vegetative to floral development [reviewed by [12]]. The coincidence of *CO *expression with light in the late afternoon in LD stabilized the CO protein resulting in up-regulation of *FT *in the late afternoon and promotion of flowering [reviewed by [13]]. In SD, *CO *was expressed predominantly in the night and CO protein was degraded and thus flowering was not promoted [reviewed by [13]].

*GI *was placed upstream of *CO *in the photoperiod pathway, as *CO *expression was reduced in *gi *mutants and up-regulated by over expression of *GI *from the cauliflower mosaic virus 35S promoter (35S) [5,14]. As expected from the regulatory hierarchy just described, the *gi *mutant had very low transcript levels of *FT *[14]. How GI might function at the molecular level to promote *CO *expression and flowering was not clear from its amino acid sequence which was predicted to form a large 1173 aa protein with no domains of known biochemical function such as DNA binding [2,5,7]. *GI *transcript cycled and accumulated to peak levels ~10 h after dawn with highest protein levels at ~12 h after lights on (Zeitgeber 12, ZT 12) in LD [2,15]. *CO *transcript was biphasic with a peak in the late afternoon in LD and a second peak persisting through the night and at dawn then falling to trough levels during much of the day [14,16]. Recently, GI and a blue light receptor FKF1 (FLAVIN-BINDING, KELCH REPEAT, F-BOX 1), related to ZTL, were shown to interact in a light-stimulated fashion and target a repressor of *CO *transcription - CYCLING DOF FACTOR 1 - for degradation by the proteasome [16-18]. Chromatin immunoprecipitation assays showed that the FKF1 and GI proteins interacted in vivo with the *CO *gene promoter supporting a nuclear role for GI in flowering [18].

Despite this remarkable progress, important questions remain about the molecular role of GI in promoting the transition to flowering and the other processes that it influences. For example, it is not clear if *GI *promotes flowering solely through GI-FKF1 interactions as *35S::GI *constructs accelerate flowering in *fkf1 *mutant plants [18] and *CO *transcript levels are reduced in *gi *mutants at all time points in both LD and SD [5,14], not only in the late afternoon in LD when GI and FKF1 interact in wild type plants [18].

Thus, our overall aim was to use an inducible GI system to ascertain if there were other previously unknown early targets (protein or transcript) of GI action that would cast light on the broader roles of GI. The approach chosen was to fuse the ligand binding domain of the rat glucocorticoid receptor (GR) to the C-terminus of GI. This would allow post-translational induction of GI activity by application of the steroid hormone Dexamethasone (DEX) [reviewed by [19]].

Previously, use of a similar post-translational steroid induction system was very productive in the search for early targets of the flowering time regulator CO [20-22]. Plants carrying a *35S::CO-GR *transgene flowered earlier than wild type in the presence of DEX [20] and 1 h of DEX treatment increased the expression of CO targets such as *FT *and *TWIN SISTER OF FT *(*TSF*) [21,22]. Furthermore, the increased transcript accumulation occurred in the presence of the translational inhibitor cycloheximide. This indicated that translation of other gene products was not needed once DEX had been applied and thus that *FT *and *TSF *were direct targets of CO.

Here we report on the characterisation of a steroid-inducible post-translational control system for GI in Arabidopsis.

## Results and Discussion

### A steroid-inducible GI fusion protein promotes the transition to flowering

We constructed transgenic *gi *lines to investigate floral induction and gene expression using a post-translationally-inducible GI protein. The transgenic lines (TG lines) were designed to express a GI protein fusion protein composed of a 277 amino acid ligand binding domain of the rat glucocorticoid receptor (GR) fused to the C-terminus of GI in a *gi *mutant background (ecotype Columbia, Col, carrying the strong *gi-2 *allele [2]). The fusion gene was expressed from the constitutive 35S promoter. The transcript and protein product of the *35S::GI-GR *construct were expected to be present throughout the day/night cycle in LD in the transgenic plants. Experiments with two other epitope tagged versions of 35S::GI supported this idea as immunoblotting with antibodies directed to these epitope tags showed there was only a slight variation in the steady state levels of those fusion proteins in total protein extracts in LD [15]. In addition, the GI protein fusions to these epitope tags were functional in that they could rescue the late flowering phenotype of *gi-2 *mutants in LD [15].

The GI-GR fusion proteins described here would be expected to be non functional in the absence of added steroid and retained in the cytoplasm, while in the presence of DEX, the fusion protein would relocate to the nucleus and be activated [reviewed by [19]]. This would provide the opportunity to test the ability of the DEX activated GI-GR fusion protein to rescue the late flowering *gi-2 *phenotype and induce gene expression.

**Figure 1:**
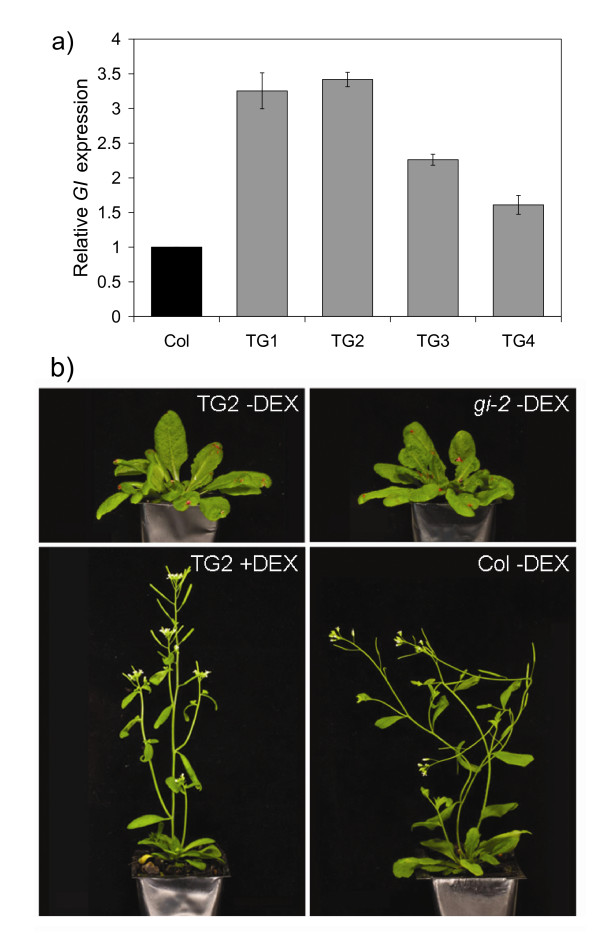
***GI *expression and flowering time phenotype in transgenic (TG) and control Arabidopsis plants under long day conditions in response to application of the steroid dexamethasone (DEX)**. a) *GI *transcript accumulation was measured using qRT-PCR. Relative transcript abundance 10 h after lights on is shown with levels normalised to *ACTIN2 *(mean +/- SD of 2 qRT-PCR runs is shown). b) Photographs of 41 day old TG2 and control plants (Col and *gi-2 *mutant plants) treated with DEX (+DEX) or control solutions (-DEX) from the time of imbibition. The pink dots on the leaves were made to assist with leaf counts.

Four independent, homozygous, single-locus insertion lines of *35S::GI-GR gi-2*, named TG1 to TG4, were generated and used for further work. As expected from a transgene expressed from the 35S promoter, total *GI *transcript accumulated to higher levels in all four TG lines compared to Col plants (Figure 1a). To test if the *35S::GI-GR *construct was functional, groups of TG, Col and *gi-2 *mutant plants were grown in LD conditions and watered either with DEX (+DEX) or control solutions (-DEX). DEX application started at seed imbibition and was repeated every 3 to 4 days after that. Photographs of 41 day old plants showed that +DEX TG plants had well-developed inflorescences, but like *gi-2 *plants, the -DEX TG plants showed no sign of flowering (Figure 1b). This indicated that DEX induction of the GI-GR fusion protein in the TG lines rescued the late flowering *gi-2 *mutant phenotype.

**Figure 2:**
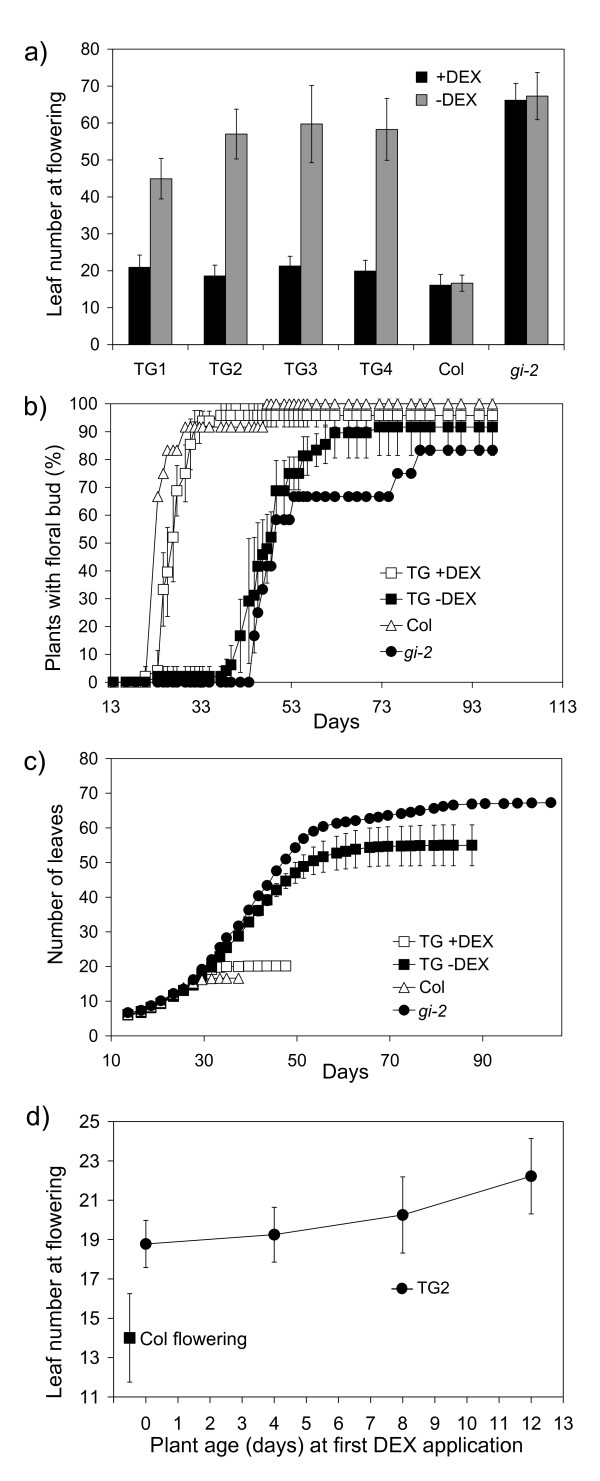
**Flowering time measurements in transgenic (TG) and control Arabidopsis plants under long day conditions in response to application of the steroid Dexamethasone (DEX)**. a-c) Plants were treated with DEX (+DEX) or control solutions (-DEX) from the time of imbibition. n = 10-12. a) Total number of leaves (rosette + cauline) at flowering. The data is presented as mean +/-SD. b) Percentage of plants showing visible floral buds and c) number of leaves developed during the life cycle. For b) and c), the data from the four TG lines is presented as mean +/- SD. d) TG2 plants were treated with DEX or control solutions every 4 days from the days shown and total numbers of leaves at the time of flowering were counted. The data is presented as mean+/-t.se; p = 0.05, n = 4-9. The flowering time of wild type Col plants is shown for comparison.

Flowering time was measured by analyzing leaf number and by counting the days from germination to flowering. The TG lines flowered earlier in the presence of DEX than in its absence using either method (Figure 2a to 2c). The results from graphing leaf counts over time demonstrated that TG and control plants produce leaves at a similar rate as the control plants in all treatments before flowering (Figure 2c), while the flowering time of Col and *gi-2 *mutant plants was not affected by DEX application (Figure 2a to 2c).

Figure 2a shows the total leaf number at the time of flowering in the presence or absence of DEX. Following DEX application, all the TG plants flowered much earlier than non-treated plants. The +DEX TG plants flowered with an average of 20.2 leaves +/- SD 2.9 while the -DEX TG plants flowered much later with an average of 55 leaves +/- SD 7.8. This is comparable to Col wild type plants which flowered with 16.1 leaves +/- SD 2.9 and *gi-2 *mutant plants which flowered at 67.3 +/- SD 6.4 leaves respectively.

One exception was the -DEX TG1 plant group which flowered with 44.9 +/- SD 5.5 leaves indicating some "leakiness" in the control of flowering by the *35S::GI-GR *construct in this transgenic line. This was unexpected as qRT-PCR of *GI *transcript levels (Figure 1a) indicated that *GI *transcript accumulated to a similar level in TG1 and TG2. It is possible that this difference between the two TG lines might be due to a slight change in the GR portion of the fusion protein that occurred only in the TG1 transgenic plant. This may have led to it being retained less well in the cytoplasm in the absence of DEX in these plants. The sub-cellular location of the GI-GR fusions could be analysed using western blotting on plant sub-cellular fractions. Unfortunately, antibodies we raised to the GI protein did not detect GI in plant extracts and a commercial antibody could not be located that would detect the GR portion in immunoblotting.

Figure 2b presents the results of the days-to-flowering measurement carried out on four TG lines and control plants. The earliest flowering group consisted of +/-DEX Col plants and more than 50% of these had flowered by 23 days. Shortly afterwards, the second group started to develop flowers. This group consisted of the TG plants treated with DEX; more than 50% of these plants had flowered by 27 days. The third group consisted of plants from the +/-DEX treatments of the *gi-2 *mutant and of the -DEX TG lines; more than 50% of these had flowered at 50 days. These groupings are similar to those seen from the leaf counts (Figure 2a).

In order to gain insight into when the TG lines first became responsive to DEX, groups of TG2 plants were grown in LD conditions and sprayed with DEX every 4 days starting with the first group where seeds were imbibed with DEX (day 0) and the last group treated from 12 days old (Figure 2d). Flowering time measurements showed that plants sprayed from day 12 onwards (flowering at an average of 22.2 leaves +/- t.se 1.2; p 0.05) were significantly later flowering than day 0 plants (18.8 leaves +/- t.se 1.9; p 0.05) (Figure 2d). This indicated that the TG2 plants were responsive to DEX within the first 8-12 days of development. In another experiment with the TG2 line, we obtained similar results and found that plants remained sensitive to DEX even when it was first applied to much older plants - at 24 days-old, an age by which wild type Col plants would have flowered (Figure 2b). These +DEX TG plants flowered with an average of 39.3 leaves +/- SD 2.1 compared to the -DEX controls which flowered at 66.2 leaves +/- SD 18.

### Induction of flowering gene expression in the transgenic lines 28 h after DEX application

**Figure 3:**
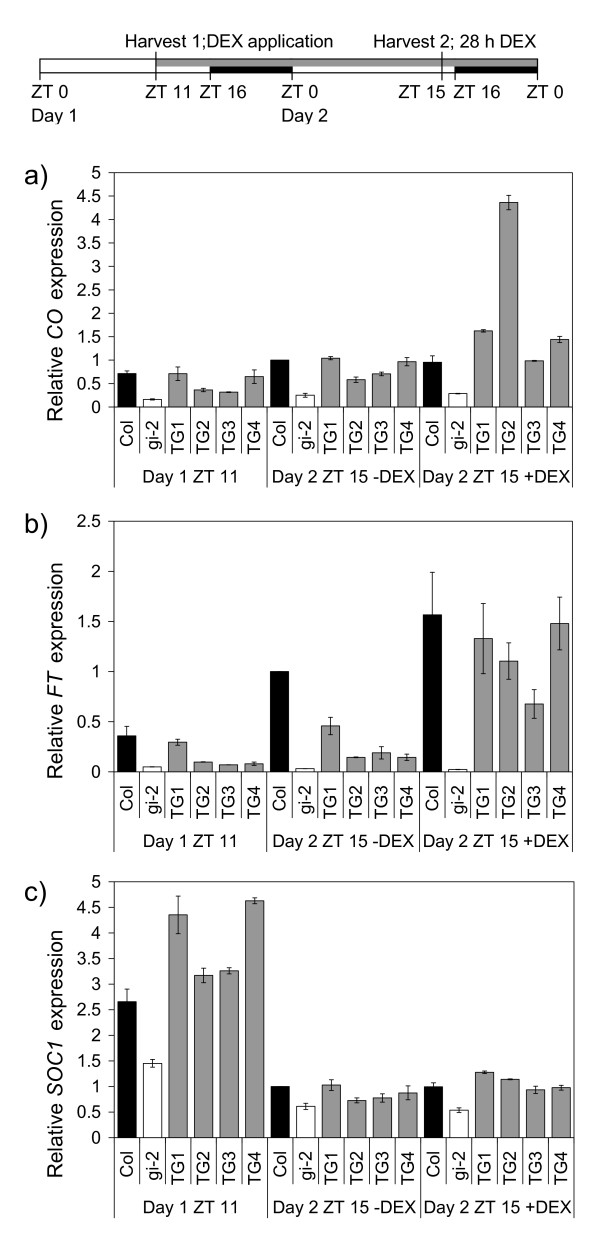
**Analysis of transcript abundance of flowering-time genes in transgenic (TG) and control Arabidopsis plants in long day conditions 28 h after DEX application**. a) *CO *b) *FT *c) *SOC1*. Relative transcript accumulation is shown at ZT 11 just prior to DEX application and at ZT15 on Day 2, 28 h after DEX was applied to 15 day-old plants growing on agar plates in LD. Plants were treated with DEX (+DEX) or control solutions (-DEX). Transcript abundance was quantified using qRT-PCR and expression levels were normalised to *ACTIN2*. The data is presented as mean +/- SD of 2 qRT-PCR runs. The black bars on the harvest scheme indicate night, the open bars indicates day and the grey bar indicates the length of treatment with DEX or control solutions, ZT0 is lights on.

Since DEX treatments led to a dramatic reduction in flowering time of the *35S::GI-GR gi-2 *mutant plants, we expected that potent flowering time activators such as *FT *would be induced by DEX application. In order to begin to investigate the effect of DEX induction of GI activity on gene expression in floral inductive LD, we used quantitative Reverse Transcriptase RT-PCR (qRT-PCR) to measure the effect on known *GI *downstream flowering-time genes, *CO*, *FT *and *SOC1*. Fifteen day-old plants from all four of the TG lines and controls grown in LD on agar plates were treated with DEX and then harvested 28 h later, 15 hours after lights on, during the late afternoon (ZT15) on Day 2 (Figure 3).

The selection of this growth regime and harvest time was an important consideration. First, as we were interested in the promotive effects of GI on flowering, we carried out the experiments in LD. Second, previous work had shown that both *FT *and *CO *gene expression cycles with high points late in the light period of LD [14]. Third, plants constitutively over-expressing *GI *had higher *CO *transcript levels throughout the day/night cycle, while they retained cyclical *FT *expression [5]. Thus, once the GI-GR fusion had been activated by DEX, it was expected that *CO *expression would be able to be analyzed at any time during the day/night cycle, and *FT *expression during the afternoon. By applying DEX at ZT11 on Day 1, when GI protein levels normally peak in wild type plants [15], we reasoned that we would be exposing the plants to the effects of GI activation both on Days 1 and 2, thus maximizing the gene expression response by ZT15 on Day 2.

The response of *FT *expression to 28 h DEX application was the strongest of the three genes (Figure 3b). The increase ranged from 2.9 to 10.1 fold. Two of the +DEX TG lines had *FT *levels as high as the -DEX Col plants. The *gi-2 *mutant expressed *FT *at 0.14 and 0.03× the level of Col plants at ZT11 and ZT15 (-DEX) respectively. Levels of *FT *were higher in the -DEX TG lines than in the *gi-2 *mutant (up to 14.2× higher), indicating some leakiness in the function of the gene construct, but still less than the levels observed in Col plants (0.15 to 0.5× Col levels at ZT15, -DEX). The good level of DEX induction of *FT *transcript accumulation was consistent with the acceleration of flowering in TG lines treated with DEX (Figure 1 and 2).

In three of the +DEX TG lines, *CO *expression rose weakly (1.4× to 1.6×), while the fourth line showed a more dramatic boost with an increase of 7.5× over the -DEX controls (Figure 3a). *CO *expression in the +DEX TG lines was higher than in Col plants at ZT15 in all cases. However, we observed high background *CO *gene expression in -DEX TG plants; the *gi-2 *mutant expressed *CO *at 0.2 and 0.3× the level of Col plants at ZT11 and ZT15 (-DEX) respectively, but expression in the -DEX TG plants was higher at 0.4 to 0.9× the level of Col plants. This high level of *CO *expression, close to wild type Col levels, was not expected as it did not correlate with the late flowering observed in the -DEX TG plants.

Slight differences between +DEX TG and -DEX TG plants were also observed for *SOC1 *expression; but there was less than a two-fold increase in the +DEX lines (1.1 to 1.6×) (Figure 3c). Background levels of *SOC1 *expression in the -DEX TG plants were high as they were similar to Col plants at ZT15. Even higher background levels were observed at ZT11. At this time point, all the -DEX TG lines had *SOC1 *expression that was higher than Col plants. The *gi-2 *mutant itself expressed moderate levels of *SOC1 *at about 0.5× that of Col plants at ZT11 and ZT15 (-DEX). This was consistent with previous reports on the effect of *gi *mutations on *SOC1 *expression in whole seedlings [23,24]. A much greater effect of *gi *mutations on *SOC1 *expression in the shoot apex would be expected as there is strong up regulation of *SOC1 *in the shoot apex in LD [23,24], but this would be greatly diluted in our experiments as we examined *SOC1 *expression in total aerial parts of young plants.

We also confirmed *GI *transcript levels in the transgenic plants were not affected by DEX application. DEX was applied at ZT8 to 21- day-old TG2 plants grown on agar plates in LD. QRT-PCR showed that *GI *transcript levels were the same in the -DEX/+DEX plants when they were compared at 4 different time points; 8 h, 16 h, 24 h or 32 h later (data not shown).

### Induction of flowering gene expression in the transgenic lines 8 and 16 h after DEX application

**Figure 4:**
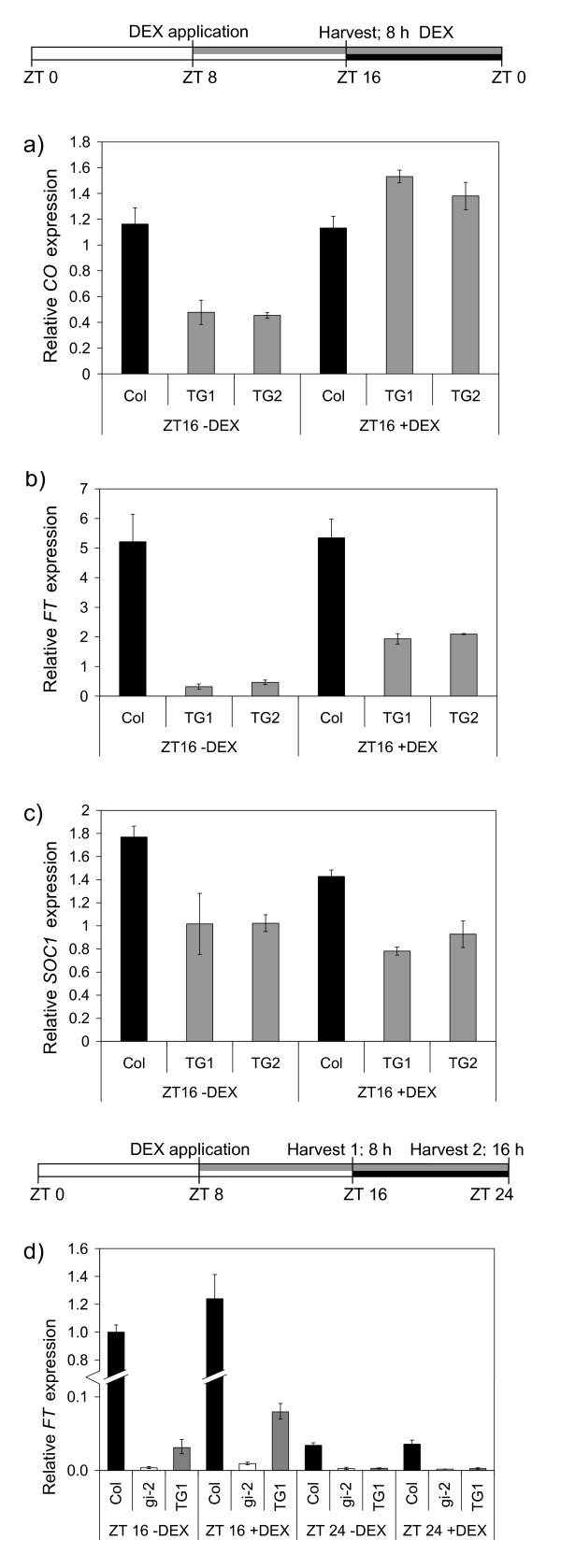
**Analysis of transcript abundance of flowering-time genes in transgenic (TG) and control Arabidopsis plants in long day conditions 8 or 16 h after DEX application**. a) *CO *b) *FT *c) *SOC1*. Relative transcript accumulation is shown 8 h after DEX was sprayed onto 15 day- old plants growing in hydroponic media. Plants were treated with DEX (+DEX) or control solutions (-DEX) at ZT8 and harvested at ZT16. d) *FT *transcript accumulation is shown either 8 h (ZT16) or 16 h (ZT24) after DEX was sprayed onto 18 day-old plants growing in hydroponic media in LD. Plants were treated with DEX (+DEX) or control solutions (-DEX) at ZT8. Transcript abundance was quantified using qRT-PCR and expression levels were normalised to *ACTIN2*. The data is presented as mean +/- SD of 2 qRT-PCR runs for a and b) and a single run for c). The black bar on the harvest scheme indicates night, the open bar indicates day and the grey bar indicates the length of treatment with DEX or control solutions, ZT0 is lights on.

Since the 28 h DEX treatment gave increases in flowering gene expression, particularly *FT*, for all TG lines (Figure 3) the DEX treatment was decreased to gain some insight into the kinetics of this induction (Figure 4). In this experiment, DEX was sprayed onto plants grown in plant growth cabinets. This was done to match the gene expression experiments to the conditions used to measure flowering time and examine if the high background levels of flowering time gene expression in the -DEX plants (Figure 3) was also observed in plants growing in these non- sterile conditions.

*FT *expression in the TG lines showed more than a 4-fold increase in +DEX TG lines 8 h after DEX application compared to -DEX TG plants (Figure 4b). The +DEX TG lines expressed *FT *to ~0.4× the level of wild type Col plants 8 h after DEX was applied. *CO *expression was increased >3× after 8 h in both the +DEX TG lines compared to -DEX treatments and was at a higher level than in Col plants (Figure 4a). *SOC1 *expression in both + DEX TG lines was not increased and it was expressed at a comparable level to the controls at ZT16 (Figure 4c) indicating that 8 h was not sufficient to alter *SOC1 *expression in these conditions.

In the -DEX TG lines, *FT *transcript levels were less than 0.1× that of wild type Col plants, *CO *transcript was 0.4× that detected in Col plants and *SOC1 *expression was 0.6× the level of Col plants. Using wild type Col as a calibrator, it appeared that the background gene expression in the -DEX TG plants was reduced when plants were grown in non sterile conditions (Figure 4) compared to on agar plates (Figure 3).

The spray assay for *FT *expression was repeated in a time course where 18 day old plants were sprayed at ZT8 and harvested 8 h later (ZT16) and 16 h later at ZT24 (Figure 4d). The *gi-2 *mutant was included to test the level of *FT *expression in this mutant when grown in non-sterile conditions and compare it to the -DEX TG plants. After 8 h of induction (at ZT16) the +DEX TG line showed a 2.6-fold *FT *induction over the -DEX TG control. The -DEX TG line expressed *FT *at 0.03× the level of Col and 8.5× the level of *gi-2*. Thus, the accumulation of *FT *in the -DEX TG line was higher than *gi-2*, but considerably less than observed in Col plants, consistent with the flowering time data. After 16 h of induction, at ZT0, *FT *levels in all genotypes tested were very low. This was expected as *FT *expression cycled even when *GI *was constitutively expressed; ZT0 was a low point in the *FT *expression cycle, coming after a period of darkness when *FT *accumulation drastically declines due to the instability of the CO protein during darkness [[5], reviewed by [13]].

### Expression of the putative flowering time gene *HAP3A *and the circadian clock gene *TOC1 *after application of DEX

Accumulation of transcript of *HEME ACTIVATOR PROTEIN 3A *(*HAP3A*) a putative flowering time regulator proposed to be positively regulated by *GI *[25] and the circadian clock gene *TOC1 *was examined. *TOC1 *transcript accumulation was previously proposed as being positively regulated by *GI *in a regulatory sub-circuit of the circadian clock [26].

**Figure 5:**
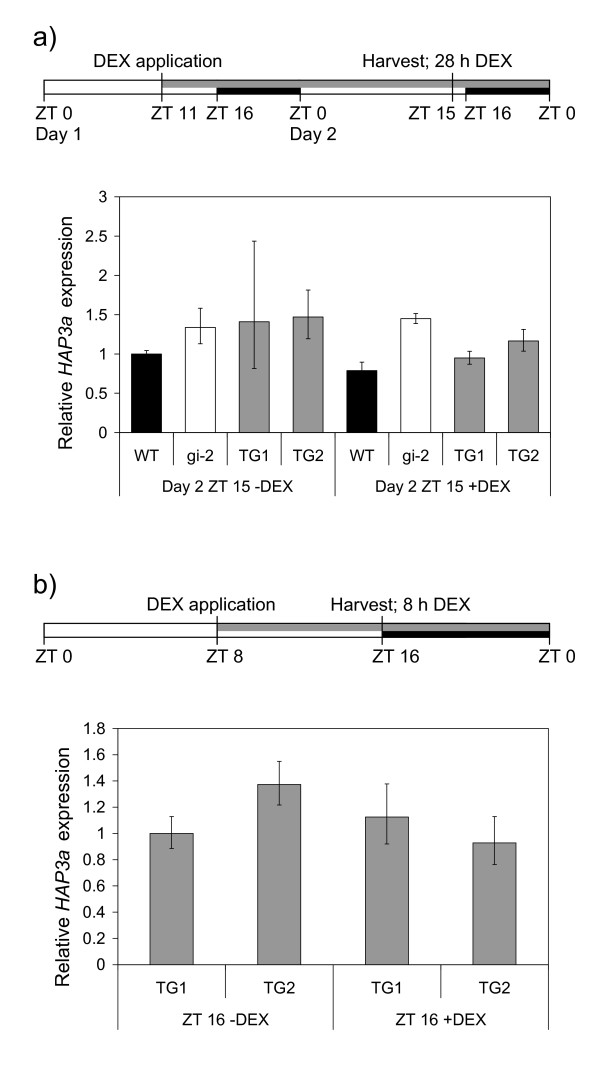
**Analysis of transcript abundance of the putative flowering-time gene *HAP3A *in transgenic (TG) and control Arabidopsis plants in long day conditions after DEX application**. a) Relative transcript accumulation is shown at ZT15 on Day 2, 28 h after DEX was applied to 15 day-old plants growing on agar plates in LD. b) Relative transcript accumulation in TG plants is shown at ZT 16, 8 h after DEX was sprayed onto 15 day-old plants growing in hydroponic media in LD. Plants were treated with DEX (+DEX) or control solutions (-DEX). Transcript abundance was quantified using qRT-PCR and expression levels were normalised to *At2g32170*. The data is presented as mean +/- SD for 3 qRT-PCR replicates. The black bars on the harvest scheme indicate night, the open bars indicate day and the grey bar indicates the length of treatment with DEX or control solutions, ZT0 is lights on.

In plants over expressing *GI *(*35S::GI*), *HAP3A *had been detected at all time points and at increased levels particularly towards the end of the day, compared to wild type plants [25]. Therefore, *HAP3A *expression was analysed either 28 h after DEX application (at ZT15), or 8 h after DEX spraying (at ZT 16), in TG and control plants. *HAP3A *expression was generally very similar across all genotypes and treatments (Figure 5). No induction of *HAP3A *expression was seen in +DEX TG lines compared to -DEX lines in either experiment.

**Figure 6:**
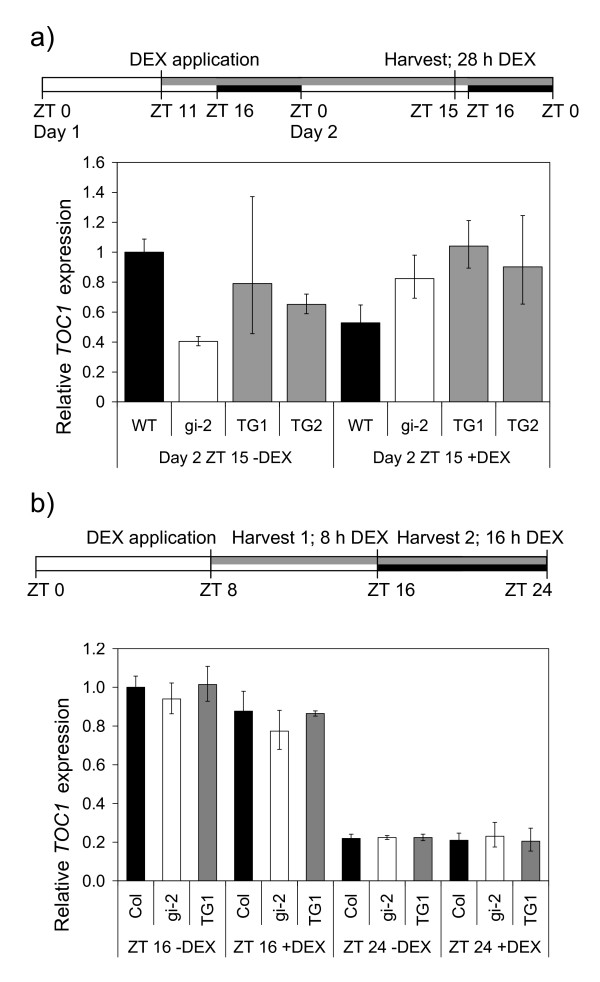
**Analysis of transcript abundance of the *TOC1 *circadian clock gene in transgenic (TG) and control Arabidopsis plants in long day conditions after DEX application**. a) Relative *TOC1 *transcript accumulation is shown at ZT15 on Day 2, 28 h after DEX was applied to 15 day-old plants growing on agar plates in LD. b) Relative transcript accumulation is shown either 8 h (ZT16) or 16 h (ZT24) after DEX was sprayed onto plants growing in hydroponic media in LD. Plants were treated with DEX (+DEX) or control solutions (-DEX). Transcript levels were normalised to *At2g32170 *in a) or *ACTIN2 *in b). The data is presented as mean +/- SD for 3 qRT-PCR replicates. The black bars on the harvest scheme indicate night, the open bars indicate day and the grey bar indicates the length of treatment with DEX or control solutions, ZT0 is lights on.

Expression of the clock gene *TOC1 *is circadian regulated and peaks in the late afternoon [27]. We tested if DEX application led to induction of *TOC1 *at ZT15 (28 h after DEX application) (Figure 6a) or at ZT16 or at ZT 24 (8 h or 16 h after application of DEX) (Figure 6b). *TOC1 *was expressed at higher levels in the evening than at dawn in wild type Col plants as expected (Figure 6b). This pattern was seen in all the genotypes including the +DEX TG line, indicating that DEX induction of GI activity had not altered the pattern of core-clock gene regulation in LD. The daily expression pattern of two other core-clock genes was also not altered by DEX application in this experiment (data not shown). *TOC1 *expression was similar across all genotypes in these experiments. Neither loss of GI activity in the *gi-2 *mutant, or induction of GI activity in the +DEX TG lines resulted in changes to *TOC1 *expression compared to Col plants (Figure 6a, b). An experiment was also performed where plants grown in liquid culture in continuous light were exposed to DEX, but again there was no change in *TOC1 *or *HAP3A *expression after 8, 16 or 24 h of DEX treatment of TG1 and TG2 plants (data not shown).

## Conclusion

DEX application to the TG lines successfully rescued the late flowering phenotype conferred by the *gi-2 *mutation. The induction of GI activity by DEX supports the idea that GI functions to promote flowering from within the nucleus as suggested by the work of Sawa *et al*. [18] and previously in transient assays when GI-reporter fusion proteins were observed in the nucleus and a nuclear-localisation region was defined [3,18]. Consistent with induction of flowering by DEX, increased transcript accumulation of the GI downstream floral promoters *CO *and *FT *was observed in TG plants after 8 h of DEX application.

CO has been proposed to trigger expression of *FT *by interacting with the HAP protein trimeric complex which binds to promoter CCAAT boxes [25,28]. *HAP3A *transcript levels were observed to increase in *GI*-over expressing transgenic plants, suggesting that GI may regulate *HAP3A *[25]. However, no induction of *HAP3A *was observed in our TG lines in the experimental time frame used here suggesting that transcript accumulation of *HAP3A *may not be directly regulated by GI.

Modeling and experimental testing of circadian clock function predicted that GI would fulfill part of a predicted "Y" function needed to stimulate *TOC1 *expression in the interlocking loop model of the circadian clock [26,29]. Experiments with the TG lines show no induction of GI activity by DEX on *TOC1 *transcript levels and no reduction in *TOC1 *levels in the *gi-2 *mutant, suggesting that this gene may not be directly regulated by GI. It is possible that we missed a transient increase in *TOC1 *expression. Some effects of *gi *mutations on *TOC1 *transcript accumulation were reported previously, but these experiments were carried out under very different light or temperature regimes from this work [6,8].

The TG plants were responsive to DEX induction of GI within the first 8-12 days of development. However, floral buds were only visible when the plants were ~27 days-old, and the +DEX TG plants were slightly later flowering than wild type Col control plants (Figure 2). The responses of the GI-GR TG plants to DEX application also were more modest that seen in *35S::CO-GR *plants. The latter responded to DEX from the time of seed imbibition and flowered significantly earlier than wild type plants in LD [20]. This suggests that there was some limitation on the activity of the GI-GR fusion protein. This contrasts with work in this laboratory with other epitope-tagged versions of GI that fully complemented the *gi-2 *mutant [15]. Unfortunately, we were not able to verify the effect of DEX on the cellular localization of the GI-GR fusion protein, as antibodies we raised to the GI protein did not detect GI in plant protein extracts, and a commercial antibody could not be located that would detect the GR portion in western blotting.

An intriguing problem encountered was that despite the -DEX TG plants being late flowering, there were often very high levels of expression of *GI *downstream genes such as *CO *in these TG lines compared with the *gi-2 *mutant. One explanation is that the leaky expression of genes such as *CO *was in tissues that were not competent to respond to it and thus *FT *expression and flowering was not strongly promoted. For example, expression of *CO *in the companion cells of the phloem using tissue specific promoters is highly floral promotive, whereas expression of *CO *in the shoot apex does not promote flowering [12,30,31].

In conclusion, the GI-GR system described here was functional in promoting flowering and allowed tests of induction of putative GI downstream genes. However, given the leaky gene expression observed and that full activity of GI-GR was not achieved, development of systems that tightly regulate the temporal and spatial control of *GI *transcript rather than a post translational system may be preferable in future work. For example, constructs that lead to induction of *GI *transcription specifically in the phloem would be interesting for further study of the effect of GI on flowering time.

## Methods

### Plant material, growth and treatments

All plant material used in this work was derived from the *Arabidopsis thaliana *L. Heynh ecotype Columbia (Col). The *gi-2 *mutant has been described previously [2]. Plants were grown under long-day conditions (16 h light/8 h dark) in controlled growth cabinets in 100 - 110 μM m^-2 ^s^-1 ^fluorescent light at 22°C. For flowering time analyses, plants were grown in soil or rockwool blocks moistened with hydroponics media [[32], without Na_2_SiO_3_] and watered every 3-4 days with 10 μM DEX 0.01% (w/v) Tween-20 or control solution, or sprayed with 30 μM DEX 0.01% (w/v) Tween-20 or control solution. Leaves were counted every 2-3 days and the time when plants were bolting was recorded. To establish the responsiveness of transgenic plants (TG) plants to dexamethasone (DEX), 30 μM DEX 0.01% (w/v) Tween-20 was first sprayed at 4, 8 or 12 days after germination (seeds for day 0 treatment were imbibed with DEX solution) on groups of TG plants grown on rockwool and thereafter repeated every 4 days and flowering time was recorded as total leaf number. The flowering time experiments were repeated with similar results.

For gene expression measurements, seeds were surface-sterilised and grown for 2-3 weeks on MS media agar plates [33] or on rockwool blocks saturated with hydroponic media. Plants grown on MS agar were wet with 30 ml 10 μM DEX 0.01% (w/v) Tween-20 solution or control solution (plate assay), while those grown on rockwool were sprayed with 30 μM DEX 0.01% (w/v) Tween-20 solution or control solution (spray assay). For both treatments, DEX was applied 2-3 weeks after germination and plant tissue was harvested before and after DEX treatment. The gene expression experiments were repeated on independently grown plants and similar results were obtained.

### Plasmids and plant transformation

The coding region of the ligand binding domain from the rat glucocorticoid receptor (*GR*) was fused to the 3'-end of the full length *GI *cDNA driven by the CaMV 35S promoter (*35S::GI-GR*). Details of the cloning procedure can be obtained from the authors. The construct was transformed into the *gi-2 *mutant background and kanamycin-resistant transformants selected. Four independent homozygous, single copy, transformed lines were used for further work. The presence and identity of the *GI-GR *gene fusion junction was confirmed in all 4 TG lines by PCR and DNA sequencing.

### RNA extraction, cDNA synthesis and qRT-PCR

For gene expression experiments RNA was extracted from 50 - 100 mg plant tissue using the RNeasy^® ^Plant Mini Kit (Qiagen) and a DNase on-column treatment was carried out during RNA extraction. RNA quality and quantity was confirmed using RNA Nano Labchips (Agilent Incorp.) analyzed on an Agilent 2100 Bioanalyzer. One-two micrograms total RNA was transcribed into cDNA with Superscript III reverse transcriptase (Invitrogen) according to the manufacturer using a (dT)_17 _primer (5'-GACTCGAGTCGACATCGATTTTTTTTTTTTTTTTT-3'). As a control for potential genomic DNA contamination the same procedure was carried out omitting the reverse transcriptase. To determine relative gene expression levels using quantitative Real Time PCR (qRT-PCR), 1 μl cDNA was used in a total reaction volume of 10 μl 1× SYBR^® ^Green PCR Master Mix (Applied Biosystems) with final primer concentrations of 0.5 μM. Each cDNA sample was analysed in triplicate qRT-PCR reactions, either once or twice, on a 7900 HT Sequence Detection system (Applied Biosystems). Relative gene expression levels were calculated using the 2^-ΔΔCT ^method [34]. The gene expression experiments were repeated on independently grown plants and similar results were obtained.

### Primers used for qRT-PCR

Primers that were used for quantification of gene expression levels were tested for amplification efficiency prior to use with a dilution series of an arbitrary cDNA sample. The following primer pairs were used for qRT-PCR; 5'-TTGCAACTCCAAGTGCTACG-3' and 5'-GCTCGAAGGAGTTCCACAAG-3' for *GI*, 5'-ACTGGTGGTGGATCAAGAGG-3' and 5'-GAATTAGGGAACAGCCACGA-3' for *CO*, 5'-CTGGAACAACCTTTGGCA AT-3' and 5'-TACACTGTTTGCCTGCCAAG-3' for *FT*, 5'-CGAAAGCTTCCTCCTGGTTA-3' and 5'-GAGTTTTGCCCCTCACCATA-3' for *SOC1*, 5'-GATTCCACGAGTTTGGGAGA-3' and 5'-CCTTAGCCATTGGGAGATCA-3' for *TOC1*, 5'-GCGTTGCCTCCTAATGGTAA-3' and 5'-ACCCTCCAACTCCCTGTACC-3' for *HAP3A*, 5'-TGCTTTTTCATCGACACTGC-3' and 5'-CCATATGTGTCCGCAAAATG-3' for *At2g32170*, 5'-CTCTCCCGCTATGTATGTCGCCA-3' and 5'-GTGAGACACACCATCACCAG-3' for *ACT2*.

## Authors' contributions

MG carried out flowering time experiments and gene expression experiments, drew the figures and helped write the manuscript, EFL carried out gene expression experiments, KD helped to produce and test the transgenic lines and to criticize the manuscript, JP conceived of the study, supervised the overall project and wrote the manuscript. All authors read and approved the final manuscript.
